# Exploring the Effect of Individual and Group Level Factors on the Level of Rural Residents’ Domestic Waste Sorting: Evidence from Shaanxi, China

**DOI:** 10.3390/ijerph191912022

**Published:** 2022-09-23

**Authors:** Jihong Zeng, Yongliang Mao, Minyue Xu, Bei Jian, Mei Qu

**Affiliations:** College of Economics and Management, Northwest A&F University, No.3 Taicheng Road, Yangling, Xianyang 712100, China

**Keywords:** personal norms, social capital, *mianzi*, domestic waste-sorting, rural China

## Abstract

Rural residents are the main agents of rural domestic waste-sorting; their level of sorting directly relates to the improvement of their rural living environment and the construction of “beautiful countryside”. Considering the data of 943 rural residents in Shaanxi Province, China, this study incorporates the factors at the individual and group levels and explores how personal norms and *mianzi*, which is the individual’s reputation and social status, at the individual level and social capital at the group level impact the level of rural residents’ domestic waste-sorting through an Ordered Probit model. The results show that personal norms and *mianzi* at the individual level play an important role in improving the level of rural residents’ domestic waste-sorting. Meanwhile, social capital (i.e., social networking, social trust, social participation, and social norms) at the group level has significant positive impacts on the level of rural residents’ domestic waste-sorting. Furthermore, *mianzi* plays a moderating role between personal norms, social capital, and the level of rural residents’ domestic waste-sorting, respectively. These findings are useful for the Chinese government to formulate a policy about enhancing the level of rural residents’ domestic waste-sorting to ease the rural environmental problem.

## 1. Introduction

Environmental problems caused by the accumulation of domestic waste have become increasingly serious issues in worldwide rural areas. Especially obvious in China, it is estimated that the total generation of rural domestic waste is about 161 million tons every year, of which nearly 25% has not been collected and treated, causing polluted groundwater, soil, and air, threatening the rural living environment, and further affecting human health seriously [1,2] It is undeniable that the level of domestic waste management is relatively lagging behind in China [1]. In addition, with the continuous advancement of the rural revitalization strategy, improving the living environment and building beautiful and livable villages has become the common vision of nearly 600 million rural residents, causing the problem of rural domestic waste pollution to need to be solved urgently. Extensive studies in the literature have confirmed that domestic waste-sorting at the source is the most effective method to solve rural environmental problems in that it is beneficial for reducing the pressure of rural domestic waste treatment, and realizing recycling and harmless domestic waste management [3,4]. The Chinese government carried out the first batch pilot areas of rural domestic waste-sorting and resource utilization demonstration work to alleviate rural environmental problems in 2017, and further emphasized the promotion of local domestic waste-sorting and resource utilization by the end of 2020 [5]. The No. 1 Central Documents over the years have emphasized the domestic waste-sorting in China’s rural areas. However, limited by the lack of a main body of rural domestic waste-sorting, these polices are difficult to effectively promote [6]. Exploring the key influencing factors of the level of rural residents’ domestic waste-sorting is important for improving the rural living environment and building beautiful villages, so as to innovate rural domestic waste-sorting policies and improve rural governance systems.

In order to improve the level of rural residents’ domestic waste-sorting effectively, many scholars have interpreted the influencing factors from different perspectives, such as internal and external factors, social and psychological factors, socio-demographic factors, and so on [7,8]. The correlation that these determinants affect intentions and actual behavior has been discussed in many studies. Zhang et al. (2018) explored residents’ waste-sorting intention and behavior by including psychological behavioral antecedents and external circumstantial factors in a theoretical research model [9]. Ao et al. (2022) identified the driving factors of rural residents’ domestic waste-sorting behavior, pointing out that social and psychological factors, including publicity and education, attitudes, subjective norms, and sense of belonging, are critical influencing factors [10]. Additional research indicated that socio-demographic factors are important for domestic waste-sorting behavior. Gender, age, education, and personal economic situation have a significant effect on waste-sorting behavior [11]. However, although the types of domestic waste-sorting behavior are well addressed, there are conflicts and contradictions among these determinates [8]. Different from previous studies, this study divides the factors into individual level and group level based on human sociality and emotionality. This classification method can resolve the conflict of theoretical viewpoints of multi-disciplines from the research logic of the interdisciplinary integration of psychology and sociology, which is the basic governance context for solving China’s rural environmental problems. Individual-level factors include pollution cognition, environmental attitude, subjective norms, perceived behavioral control, and so on. Most studies suggested that residents with a positive environmental attitude are more likely to engage in recycling [11,12]. Some scholars also pointed out that the waste-sorting publicity and education are the most significant predictors of rural domestic waste-sorting behavior [10]. Further, the effectiveness of residents’ behavior depends on their internal moral guidelines, namely, personal norms [13]. Guo et al. (2020a) indicated that personal norms play a more significant role in influencing rural residents’ pro-environmental behavior rather than mandatory policy [14]. Based on the theory of planned behavior (TPB) and normative activation models (NAM), Shen et al. (2020) demonstrated that personal norms play a mediating role between subjective norms and perceived behavior control on rural residents’ domestic waste-sorting intentions, respectively [12]. In addition, scholars found that Chinese rural sociality is a relationship structure combining kinship, blood, and geography under the action of the acquaintance mechanism [15]. The group-level factors in rural acquaintance society have a strong influence on rural residents’ behavior and even exceed the formal system to a certain extent when formal institutions and resources are unavailable. Rural residents’ behavior has obvious group characteristics, causing them to be more willing to communicate and interact with others in the same relationship network [16,17]. It means that an informal system such as social capital easily affects rural residents’ behavior through improving their sense of belonging and trust, which could promote rural residents’ level of waste-sorting behavior [8,18,19,20]. Jia and Zhao (2020) pointed out that social networking, institutional trust, social participation, and social norms in social capital have significantly improved the level of rural residents’ household waste classification [21]. More importantly, the social structure of rural China is linked with a range of Chinese cultural traditions, such as *mianzi* at the individual level [22]. *Mianzi* is rooted in Chinese social networks and is important to Chinese people; it is the individual’s self-image and social position recognized by others [23,24]. In general, a typical Chinese person is sensitive to how they appear to others and is concerned with saving face for himself (or herself) and others. Especially in China’s rural communities, rural residents pay more attention to the recognition and evaluation of others within the group, as well as their appearance to others [25]. Therefore, it is reasonable to assume that they tend to be governed by *mianzi* when involved in collective activities and traditional socializations.

It is noteworthy that the existing studies have focused on individual-level or group-level factors on rural residents’ pro-environmental behavior, while the research considering the comprehensive impact of these two levels of factors on rural residents’ domestic waste-sorting is limited. To fill the above knowledge gaps, this study incorporates the factors at the individual and group level into a comprehensive analysis framework, in order to provide a multi-dimensional thinking perspective for the research on the domestic waste management in China’s rural areas. This study aims to provide an empirical test for the cross-disciplinary integration of rural waste-sorting management and a reference for the government to formulate policies to enhance rural residents’ level of domestic waste-sorting. Hence, the Ordered Probit model will be employed to explore the effect of individual and group influencing factors on the level of rural residents’ domestic waste-sorting. It is ideal for the analysis of variables with multi-class discretization and internal ordering, and can be well applied to the study.

The remainder of this study Is framed as follows: Section 2 is the conceptual framework and research hypotheses; Section 3 focuses on the research methodology given the details of data collection in this study; Section 4 and Section 5 highlight the results of the data analyses and a discussion of the findings, respectively. Section 6 and Section 7 provides our policy implications and conclusions.

## 2. Conceptual Framework and Research Hypotheses

### 2.1. Personal Norms

As an intrinsic moral guide for one’s behavior, personal norms regulate individual behavior through intrinsic pride and guilt [2,13]. According to NAM, individuals with stronger personal norms are more likely to implement their needs of behaviors in daily life [26]. Some studies have shown that personal norms are the main predictor for individuals to participate in pro-environmental behaviors [27,28]. Based on a sense of environmental moral responsibility, rural residents will possibly be proud of their actions about improving rural waste pollution problems, while they will possibly be guilty for their actual actions about violating their personal norms. Therefore, rural residents with stronger personal norms will possibly adopt waste-sorting behavior due to their sense of environmental moral responsibility and attempting to build a better living environment. Based on the above analysis, this study proposes the following hypothesis:

**Hypotheses** **1** **(H1).** *Personal norms have a significant positive impact on the level of rural residents’ domestic waste-sorting.*

### 2.2. Social Capital

Social capital was systematically studied by Putnam in the 1990s, and then gradually became a popular research field in sociology [19,29]. Social capital is regard as “soft capital” accumulated in social network relationships to achieve individuals’ or organizations’ goals, and promotes residents’ relationships and the health development of the community [30,31]. Most scholars claim that social networking, social trust, social participation, and social norms in social capital could effectively avoid the “free rider” phenomenon in the supply of rural public goods and implement collective cooperation [20,32,33,34].

According to the Network Embedded Theory, individuals cannot act independently from social situations [35]. Social networking refers to the interpersonal relationship based on individuals’ connections [36]. Jia and Zhao (2020) suggested that due to social interactions between rural residents with others based on a differential pattern of villages, this social pattern could reduce rural residents’ uncertainty of behavioral decision-making [21]. Meanwhile, social trust, as a vital component of social capital, is considered as an informal rule generated by group interaction, which encourages people to comply with common value guidelines [37]. Chen et al. (2021) found that there is an important relationship between social trust and rural residents’ participation of environmental management because social trust could effectively avoid “free-ride” phenomenon about rural public affairs [33]. In addition, rural residents take part in rural public affairs such as waste-sorting management, reflecting their rights awareness and degree of participation, which could enhance their belonging and valuation of the village [38]. Social norms refer to the behavioral rules, regulations, customs, and value standards in a common group, which could reinforce people’s sense of moral responsibility to urge them to act on common rules and standards [14,39]. Shi et al. (2019) suggested that rural residents could make more rational decisions because of a better social system and higher social norms in rural society [40]. Considering the above, the following hypotheses are proposed:

**Hypotheses** **2** **(H2).** *Social networking has a significant positive impact on the level of rural residents’ domestic waste-sorting.*

**Hypotheses** **3** **(H3).** *Social trust has a significant positive impact on the level of rural residents’ domestic waste-sorting.*

**Hypotheses** **4** **(H4).** *Social participation has a significant positive impact on the level of rural residents’ domestic waste-sorting.*

**Hypotheses** **5** **(H5).** *Social norms have a significant positive impact on the level of rural residents’ domestic waste-sorting.*

### 2.3. Mianzi

In a Chinese context, *mianzi* is a perceived social position that is judged by others, and represents one’s social image. *Mianzi* relates rural residents’ actions closely in Chinese rural areas based on the acquaintance social structure; they attempt to improve social prestige through gaining approval and saving face [24,41]. The former aims to improve others’ evaluations about the self through enhancing social prestige [42], while the latter means that people maybe lose their positive appearance by feeling ashamed or guilty due to their inappropriate behavior in social interactions, causing them to try their best to save face [41,43]. For example, if others implement domestic waste-sorting, rural residents are considered not to fulfil their social responsibility if they do not do the same thing, which affects their image, even “*mei mianzi*”, and causes them to be embarrassed by the shame [22]. However, if rural residents actively participate in the domestic waste-sorting, they are more likely to obtain honorary titles such as “clean rural residents” and “civilized and hygienic demonstration households”, and thus save face. This sense of satisfaction will in turn enhance the pleasure and pride of rural residents’ domestic waste-sorting, prompting rural residents to improve the level of domestic waste-sorting.

*Mianzi*, as an important mobilizing force for rural residents’ actions, can amplify their pride and guilt about whether they participate in pro-environmental public affairs [44]. The impact of *mianzi* on rural residents’ pro-environmental actions will reinforce a higher social capital in an acquaintance society structure. Rural residents with higher *mianzi* maybe attach importance to the role of social capital in regulating their pro-environmental behavior because they pay more attention to social resources and the relation network around them, whereas rural residents with lower *mianzi* maybe do not perform these actions because they are paying little attention to the reputation loss. It is reasonable to assume that the rural residents tend to be governed by *mianzi* when involved in domestic waste-sorting. The following hypotheses are proposed:

**Hypotheses** **6** **(H6).** *Mianzi has a significant positive impact on the level of rural residents’ domestic waste-sorting.*

**Hypotheses** **7** **(H7).** *Mianzi plays a moderating role between personal norms and the level of rural residents’ domestic waste-sorting.*

**Hypotheses** **8** **(H8).** *Mianzi plays a moderating role between social capital and the level of rural residents’ domestic waste-sorting.*

**Figure 1:**
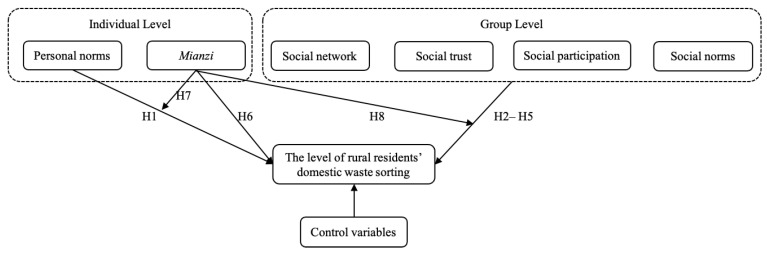
Conceptual framework and research hypotheses in the study.

As shown in Figure 1, this study constructed a conceptual framework to represent the relationship between individual factors (personal norms, *mianzi*), group factors (social networking, social trust, social participation, and social norms), and the level of rural residents’ domestic waste-sorting based on the above empirical analysis. 

## 3. Methodology

### 3.1. Data Collection

The data used in this study are from the rural resident’s household survey in seven cities of Xi’an, Xianyang, Baoji, Yan’an, Yulin, Ankang, and Hanzhong in Shaanxi Province, China in 2021. Shaanxi is an important agricultural province in China. At the end of 2020, the rural population in Shaanxi reached 14.77 million, accounting for 37.34% of the province’s total population [45]. As early as 2008, the Shaanxi government started environmental protection work in rural areas, and will continue to intensify its efforts to classify and control rural waste and carry out pilot projects for the increase of rural domestic waste-sorting in the future. The main contents of the questionnaire include: the basic information of rural residents’ households, the basic information of rural residents’ agricultural production, the generation and disposal of rural residents’ domestic wastes, the cognition of rural residents’ domestic waste disposal, the measurement of rural social capital, and so on. Before the formal survey, the authors conducted a pre-survey in Yangling District to ensure that the questionnaire was valid and usable. Afterward, a combination of hierarchical sampling and random sampling was used to select sample rural residents for a face-to-face questionnaire interview in the seven cities. As a result, a total of 1016 questionnaires were collected, and 943 valid questionnaires were obtained after excluding invalid questionnaires, with an effective rate of 92.81%.

### 3.2. Variable Selection and Statistical Description

The variable selection and statistical description used in the survey are shown in Table 1. The explained variable in this study is the level of rural residents’ domestic waste-sorting. The indicator was measured by the question, “The actual situation of your domestic waste-sorting”. The scoring standard is as follows: do not sort = 1, sort into two categories = 2, sort into three categories = 3, sort into four categories = 4.

Personal norms, *mianzi* at the individual level, and social capital (i.e., social networking, social trust, social participation, and social norms) in the group level are selected as the explanatory variables. To measure these variables, these variables were adjusted appropriately to suit the present research context on the basis of previous research. Respondents were asked to show their views and evaluate these items using a five-point Likert scale (labelled end-points of 1 = “completely disagree” and 5 = “completely agree”).

### 3.3. Model

The statistical software Stata 15.0 was employed for empirical analysis in this study. Since the explained variable in this study is ordered and discrete, this study uses the Ordered Probit model to analyze the influencing factors of the level of rural residents’ domestic waste-sorting. Based on the theoretical analysis, we construct a model of influencing factors on the level of rural residents’ domestic waste-sorting. The model form is as follows:

(1) $$Y^{*}{=\beta }_{0}{+\beta }_{1}{\;\mathrm{PN}+\beta }_{2}{\;\mathrm{SC}+\beta }_{3}{\;\mathrm{MZ}+\beta }_{4}\;\mathrm{Control}+\varepsilon$$

In the Formula (1), Y* represents the level of rural residents’ domestic waste-sorting, which is defined as an ordinal variable from 1 to 4, *β_i_* is the parameter to be estimated, *PN* represents personal norms, *SC* represents social capital, *MZ* represents *mianzi*, control variables are added at the same time, and *ε* is the random disturbance term. The selection model of the explained variable *Y* can be constructed by using latent variables:

(2) $$Y=\{\begin{array}{c} 1=\mathrm{do}\;\mathrm{not}\;\mathrm{sort},\;\;\;\;\;\;\;\;\;\;\;\;\;\;\;\;\;\;\;Y^{*}\leq \mu_{1}\\ 2=\mathrm{two}\;\mathrm{categories},\;\;\;\;\;\;\mu_{1}<Y^{*}\leq \mu_{2}\\ 3=\mathrm{three}\;\mathrm{categories},\;\;\;\mu_{2}<Y^{*}\leq \mu_{3}\\ 4=\mathrm{four}\;\mathrm{categories},\;\;\;\;\;\mu_{3}<Y^{*}\;\;\;\;\;\;\;\;\;\;\; \end{array}$$

In the Formula (2), *µ*_1_*, µ*_2_*,* and *µ*_3_ are the three demarcation points of Y*, which separate the level of rural residents’ domestic waste-sorting. When Y* ≤ µ_1_, that is, *Y* = 1, it means that rural residents do not sort domestic waste at all; when *µ*_1_ < Y* ≤ µ_2_, that is, *Y* = 2, it means that the recyclable waste is sorted; when *µ*_2_ < Y* ≤ µ_3_, that is, *Y* = 3, it means that rural residents continue to sort kitchen waste on the basis of sorting recyclable waste; and when *µ*_3_ < Y*, that is, *Y* = 4, it means that rural residents further sort hazardous waste on the basis of sorting recyclable and kitchen waste.

## 4. Results

### 4.1. Socio-Economic Characteristics of Respondents

The detailed socio-economic characteristics of respondents are shown in Table 2. Of the 943 respondents, 608 are male, and 335 are female. Most of the respondents are over 40 years old (*n* = 880, 93.32%); this is consistent with the “village-hollowing” phenomenon in rural China, whereby the young work outside the village, and the older people and children are left behind in the rural areas [51]. In addition, the percentage of rural residents with political status (*n* = 62, 6.57%) among the respondents is relatively small. Most respondents have primary school (*n* = 299, 31.71%) and junior high school (*n* = 336, 35.63%) education. The percentage of rural residents with senior high school education or secondary technical school and above is only 22.48%. The annual household income of most respondents is less than 40,000 CNY (*n* = 311, 32.98%) and between 40,001 and 80,000 CNY (*n* = 297, 31.49%). The survey of this study is basically consistent with the reality of rural Shaanxi Province, so the sample data are representative to a certain extent.

### 4.2. Empirical Results

Before estimating the Ordered Probit model, considering the possible internal correlation between the variables, we use the variance inflation factor (VIF) to test the multicollinearity between the explanatory variables. When VIF is more than 3, there is a certain degree of collinearity among explanatory variables; when VIF is more than 10, there is serious collinearity among explanatory variables. The results show that the maximum VIF is 1.50, and the average is 1.26, indicating that the possibility of multicollinearity between explanatory variables is small, which can meet the research requirements of this study. The benchmark regression is carried out on Formula (1) without considering the moderating effect of *mianzi* firstly. We obtained model (1) and (2) by introducing personal norms, social capital, and *mianzi* into the model, respectively. Secondly, the control variables are introduced into the model, and model (3) and (4) are obtained. Table 3 shows that Pseudo R^2^ of model (4) is 0.096, which is higher than models (1)–(3), indicating that it is meaningful to investigate the effects of core explanatory variables on the level of rural residents’ domestic waste-sorting at the same time. Therefore, the following empirical analysis is mainly based on the regression results of model (4). Meanwhile, we conduct a marginal effect analysis based on model (4), so as to intuitively judge the improvement effect of the variables on the level of rural residents’ domestic waste-sorting. Table 4 shows the results.

From the empirical results of model (4) in Table 3, we can see that personal norms have a significant positive impact on the level of rural residents’ domestic waste-sorting at a 1% significant level. The marginal effect in Table 4 shows that personal norms increase the level by one unit, the rural residents not sorting domestic waste can decrease it by 1.90%, and rural residents sorting into three categories and four categories can increase it by 2.20% and 1.60%, respectively. Hypothesis 1 was verified here.

Social capital significantly affects the level of rural residents’ domestic waste-sorting. Social networking significantly and positively influences the level of rural residents’ domestic waste-sorting at a statistically significant level of 5%. The marginal effect further shows that the social network of rural residents increases by one unit, the rural residents not sorting domestic waste can decrease it by 1.70%, and sorting into three categories and four categories can increase it by 1.90% and 1.40%, respectively. It shows that the closer the communication between village cadres and respected rural residents, the higher the level of rural residents’ waste-sorting behavior. Social trust has a positive impact on the level of rural residents’ domestic waste-sorting at the 5% significance level. The social trust increases by one unit, the rural residents not sorting domestic waste can decrease social trust by 1.60%, and rural residents sorting into three categories and four categories can increase it by 1.80% and 1.30%, respectively. Social participation has a significant positive impact on the level of rural residents’ domestic waste-sorting at a 1% significance level, indicating that the more frequent the participation in environmental protection affairs organized by the village, the higher the level of rural residents’ domestic waste-sorting. The results of the marginal effect of social participation show that for each unit increase in the social participation, the rural residents not sorting domestic waste will decrease it by 2.70%, and sorting into three categories and four categories will increase it by 3.10% and 2.20%, respectively. Social norms significantly and positively influence the level of rural residents’ domestic waste-sorting at the 1% statistical level. The marginal effect shows that social norms increase by one unit, the probability of rural residents not sorting decreases by 2.60%, and sorting into three categories and four categories can increase it by 3.00% and 2.20%, respectively. Hypotheses 2–5 were verified.

*Mianzi* positively impacts the level of rural residents’ domestic waste-sorting at the 1% significance level. For each unit of increase in the *mianzi*, the rural residents not sorting can decrease by 1.50%, and rural residents sorting into three categories and four categories can increase by 1.70% and 1.20%, respectively. It shows that rural residents with higher *mianzi* will gain the appreciation of others and maintain their own image actively by improving the level of rural residents’ domestic waste-sorting. Hypothesis 6 was verified here.

For control variables, age has a significant negative impact on the level of rural residents’ domestic waste-sorting at the statistical level of 5%, while the waste-sorting facility significantly and positively influences it at a 1% significance level, indicating that the existence of waste-sorting facilities significantly promotes the level of rural residents’ domestic waste-sorting. However, other control variables had no statistically significant impact on the level of rural residents’ domestic waste-sorting. The reason may be that the behavior of rural residents to dispose of domestic waste is still largely affected by their own habits and convenience, and the impact of external factors is relatively small [52].

To further test the robustness of the results, we introduce the explanatory variables and control variables into the Ordered Logit model to reduce the possibility of biased estimates from the same model estimates. The results as shown in Table 3 show that the estimated effects of the core explanatory variables are basically consistent with the regression results of model (4), indicating that the results of this study are relatively robust.

### 4.3. The Moderating Effect of Mianzi

For rural residents with different levels of *mianzi*, the impact of personal norms and social capital on their level of domestic waste-sorting may be different. We use the average value of *mianzi* as the grouping standard to conduct a group study on all the samples [53]. Treat those above the average as high *mianzi*, and the below as low *mianzi*. Additionally, compare the significant changes of coefficients of different groups to examine the effect of moderator variables by performing Ordered Probit regression, respectively.

The results in Table 5 show that personal norms, social participation, and social norms are all significant and positive coefficients in all groups of regressions, indicating that the moderating effect of *mianzi* on the influence of personal norms, social participation, and social norms on the level of rural residents’ domestic waste-sorting is not significant. However, social networking and social trust are not significant in the regression of model (6), but are significant in the regression of model (7) and the coefficient is positive, indicating that *mianzi* plays a positive moderating role in the relationship between social networking, social trust, and the level of rural residents’ domestic waste-sorting. Therefore, Hypothesis 8 is partially verified.

## 5. Discussion

**Figure 2:**
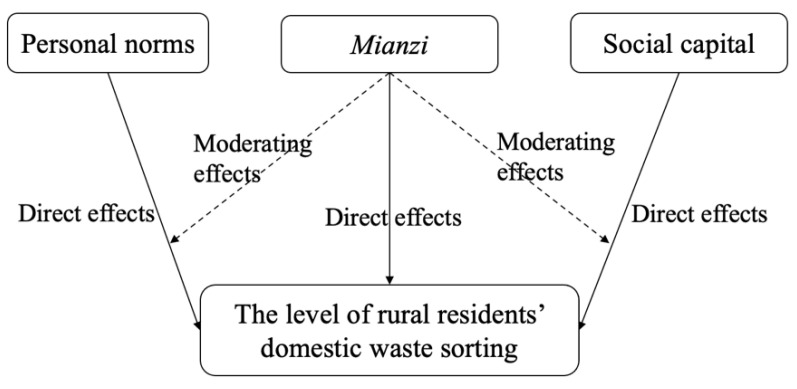
Effects diagram.

From the empirical results, we can find that the personal norms have a significant positive impact on the level of rural residents’ domestic waste-sorting (see Figure 2). When rural residents realize that they are responsible for the rural living environment, they will further subdivide domestic waste, which is consistent with previous research results. Shen et al. (2020) pointed out that attitudes, perceived behavior control, and personal norms all directly and positively affect rural residents’ intention to sort household waste, but personal norms have the greatest direct impact [12]. We found that rural residents with a higher frequency of social interaction, higher social trust, active participation in public affairs of rural waste management, and more compliance with common village guidelines have higher levels of domestic waste-sorting. This is may be rural residents with larger social networks and closer communication with others can reduce the uncertainty of behavioral decision-making and improve the level of domestic waste-sorting. The higher trust in the surrounding residents, the more they believe in the benefits of domestic waste-sorting, and the negative effects of non-sorting spread by others, thereby improve the level of rural residents’ domestic waste-sorting. Additionally, our results suggest that rural residents who participate more frequently in public affairs such as rural environmental governance will have a stronger sense of belonging to the village and identify with rural environmental governance, in order to improve the level of domestic waste-sorting. Moreover, the group members usually form a common code of conduct and value standards for the sorting of domestic waste in the whole village. Rural residents will abide by the common rules and standards of and make measures that are in line with the collective interests of the entire village. Therefore, the stronger the social norms, the stronger the willingness of rural residents to participate in the domestic waste-sorting will be, and the higher the level of domestic waste-sorting will be. The aforementioned results further validate the research results of scholars such as Jia and Zhao (2020) [21].

Figure 2 established the moderating effects of *mainzi* intuitively. We found that rural residents with higher *mianzi* are more likely to improve the level of domestic waste-sorting. The possible explanation is that rural residents with higher *mianzi* are more willing to pay attention to other’s opinions and evaluations, care about their image in the hearts of others, and improve their daily behavior to obtain honorary titles such as “clean rural residents” and “civilized and hygienic demonstration households” [41]. Therefore, rural residents with a strong sense of *mianzi* will show their attitude towards environmental protection by improving the level of domestic waste-sorting, so as to gain recognition and praise from others, and gain more approval from others [25]. When the rural residents’ *mianzi* is higher, the greater the level of social network and social trust is, the stronger the binding force on the level of rural residents’ domestic waste-sorting.

Further, the older the rural residents are, the lower the sorting level may be. This may be that the older generation of rural residents are more deeply affected by the habit of mixed treatment of domestic waste, and it is difficult for them to understand the harm of mixed treatment of domestic waste to human health, and thus are less likely to accept the concept that domestic waste needs to be sorted [8]. Additionally, the existence of waste-sorting facilities is conducive to improving the level of rural residents’ domestic waste-sorting. If pro-environmental activities, such as domestic waste-sorting, are considered to be an inconvenience, the government should introduce new policies or systems to decrease inconvenience cost by improving waste-sorting infrastructures [25,54].

There are still deficiencies in this study, which need to be further explored and improved. We discuss the factors affecting the level of rural residents’ domestic waste-sorting from the perspective of the combination of individual and group levels, which can effectively fill the knowledge gaps of previous research to a certain extent. However, the level of rural residents’ domestic waste-sorting is not only affected by these factors. The role of other factors cannot be ignored, such as government environmental regulation policies. Although this study has discussed the influence of the government in the construction of rural waste-sorting facilities in control variables, the role of the government is much more than that [55]. Especially in China, with the implementation of the rural revitalization strategy, the launch of the five-year action to improve the rural living environment, and the emphasis on rural environmental governance in the 14th Five-Year Plan, policies will play a more dominant role in rural waste-sorting [10]. Therefore, factors in this regard should be taken into account in the future, and the domestic waste-sorting behavior needs to be placed in a comprehensive social-ecological system framework for investigation and comprehensive explanation.

## 6. Policy Implications

Based on the above analysis, we can propose the following policy implications. First, continue to improve the channels and content of rural domestic waste-sorting publicity and education, and improve rural residents’ awareness of moral responsibility, thereby enhancing the knowledge of domestic waste pollution and its hazards. Second, the regulation and restraint mechanism of social capital should be established to create a village order in which rural residents actively participate in the domestic waste-sorting. The frequency of communication and information transmission between rural residents needs to be strengthened in daily life. Rural residents should be encouraged to actively participate in various collective activities. Third, it is important to construct a reward and punishment mechanism for rural domestic waste-sorting by taking advantage of rural residents’ emphasis on *mianzi*, and promote rural residents to improve the level of domestic waste-sorting through good public opinion. Finally, we cannot ignore the role of the government in the construction of rural domestic waste-sorting facilities. Rural waste-sorting facilities are the basic guarantee of implementing domestic waste-sorting. The waste-sorting facilities in rural China need to be further improved.

## 7. Conclusions

Considering the survey data of 943 rural residents in rural areas of Shaanxi Province, this study constructs a conceptual framework of the level of rural residents’ domestic waste-sorting at the individual level and group level, and uses the Ordered Probit model to discuss the impact of personal norms and *mianzi* at the individual level and social capital at the group level of rural residents’ domestic waste-sorting and the moderating effect of *mianzi*. The conclusions are as follows: personal norms and *mianzi* at the individual level have a significant positive impact on the level of rural residents’ domestic waste-sorting. Rural residents with stronger personal norms will have a sense of joy and pride when sorting domestic waste, thereby improving the level of domestic waste-sorting. Meanwhile, social networking, social trust, social participation, and social norms in social capital at the group level show a significant positive impact on the level of rural residents’ domestic waste-sorting. In addition, *mianzi* has a significant positive moderating effect on the relationship between social networking, social trust, and the level of rural residents’ domestic waste-sorting.

## Figures and Tables

**Table 1:** Variables selection and statistical description.

Variables	Description	Mean	Standard Deviation	References
**Explained variable**				
The level of rural residents’ domestic waste-sorting	The actual situation of your domestic waste-sorting: do not sort = 1, sort into two categories (recyclable, others) = 2, sort into three categories (recyclable, kitchen waste, and others) = 3, sort into four categories (recyclable, kitchen waste, harmful and others) = 4	2.392	0.812	MOHURD (2019) [46]
**Explanatory variables**				
Personal norms	I am obliged to participate in the sorting and disposal of domestic waste and clearing it at designated locations.	3.975	0.993	Guo et al. (2020a) [14]; Guo et al. (2020b) [26]
	I should maintain the cleanliness of the village.	4.098	0.815	
	Every farmer is responsible for the environmental pollution of domestic waste.	4.159	0.813	
Social network	Frequency of communication with your close friends.	4.171	0.863	Shi et al. (2018) [47]; Yang (2018) [48]
	Frequency of communication with village cadres.	2.660	0.923	
	Frequency of communication with respected rural residents.	2.863	1.010	
Social trust	Degree of trust in your neighbors.	3.929	0.711	He et al. (2015) [49]; Zhao and Dong (2019) [50]
	Degree of trust in rural residents with high morals.	3.429	0.861	
	Degree of trust in your close friends.	4.119	0.690	
Social participation	Your participation in environmental protection affairs in the village.	3.783	0.958	Jia and Zhao (2020) [21]; Shi et al. (2018) [47]
	Your participation in waste collection activities.	3.831	0.958	
	Your participation in the election of village cadres.	3.196	1.487	
Social norms	Village rules and regulations require me to actively participate in the domestic waste-sorting, and I will.	4.094	0.930	Jia and Zhao (2020) [21]; Shi et al. (2019) [40]
	Neighbors think that I should actively participate in the domestic waste-sorting, and I will.	3.791	0.872	
	The elites or capable people in the village think I should actively participate in the domestic waste-sorting, and I will.	3.618	0.854	
*Mianzi*	Compared to other people, I pay more attention to my social appearance in daily life.	3.323	1.091	Tang et al. (2019) [25]
	I am very concerned about the opinions and evaluations of others.	3.318	1.108	
	If others sort the domestic waste, my failure to sort it will affect my image in the eyes of others.	3.471	1.069	
	Pollution of the environment will make me punished and lose face.	3.118	1.275	
	I attach importance to the honorary titles such as “clean rural residents” and “civilized and sanitary demonstration households.”	2.952	1.218	

**Table 2:** Socio-economic characteristics of the respondents (*n* = 943).

Item	Response	Frequency	Percent
Gender	Male	608	64.48
	Female	335	35.52
Age	Less than 20	0	0.00
	21–40	63	6.68
	41–60	492	52.17
	60 above	388	41.15
Political status	Party member	62	6.57
	Non-party member	881	93.43
Education	No educational experience	96	10.18
	Primary school	299	31.71
	Junior high school	336	35.63
	Senior high school or secondary technical school	205	21.74
	Undergraduate and above	7	0.74
Annual household income	Below 40,000	311	32.98
	40,001–80,000	297	31.49
	80,001–120,000	215	22.80
	120,001–160,000	64	6.79
	Above 160,000	56	5.94

**Table 3:** Estimation results.

Variables	Model (1)	Model (2)	Model (3)	Model (4)	Ordered Logit
Personal norms	0.073 *	0.085 **	0.101 **	0.110 ***	0.201 ***
	(0.038)	(0.038)	(0.039)	(0.040)	(0.070)
Social network	0.069 **	0.053	0.109 ***	0.099 **	0.200 ***
	(0.035)	(0.036)	(0.038)	(0.039)	(0.069)
Social trust	0.067 *	0.076 *	0.082 **	0.092 **	0.203 ***
	(0.040)	(0.041)	(0.041)	(0.042)	(0.075)
Social participation	0.218 ***	0.159 ***	0.188 ***	0.157 ***	0.303 ***
	(0.033)	(0.034)	(0.037)	(0.038)	(0.067)
Social norms	0.189 ***	0.188 ***	0.153 ***	0.154 ***	0.298 ***
	(0.033)	(0.033)	(0.034)	(0.035)	(0.065)
*Mianzi*		0.112 ***		0.087 ***	0.154 ***
		(0.020)		(0.021)	(0.036)
Gender			−0.043	−0.028	−0.012
			(0.076)	(0.077)	(0.135)
Age			−0.007 **	−0.007 **	−0.011 *
			(0.004)	(0.004)	(0.006)
Education			−0.071	−0.066	−0.121
			(0.045)	(0.045)	(0.083)
Political status			0.171	0.178	0.292
			(0.152)	(0.152)	(0.282)
Annual household income			−0.016	−0.018	−0.051
			(0.035)	(0.036)	(0.063)
Total resident population			0.042 *	0.024	0.042
			(0.025)	(0.026)	(0.046)
Policy understanding			−0.045	−0.053	−0.105 *
			(0.035)	(0.035)	(0.063)
Environmental awareness			0.047	0.036	0.061
			(0.040)	(0.041)	(0.071)
Hazard perception			0.017	0.005	0.019
			(0.035)	(0.035)	(0.063)
Waste-sorting facility			0.547 ***	0.481 ***	0.991 ***
			(0.092)	(0.094)	(0.166)
Log Likelihood	−1054.900	−1033.393	−1024.751	−1010.739	−995.253
LR (P > chi^2^)	135.080 ***	169.040 ***	195.380 ***	214.350 ***	245.320 ***
Pseudo R^2^	0.060	0.076	0.087	0.096	0.110
Observations	943				

Note: *, **, and *** donate a statistical significance at the 10%, 5%, 1%, respectively.

**Table 4:** Marginal effect of Ordered Probit model.

Variables	Sorting Level = 1	Sorting Level = 2	Sorting Level = 3	Sorting Level = 4
Personal norms	−0.019 ***	−0.019 ***	0.022 ***	0.016 ***
	(0.007)	(0.007)	(0.008)	(0.006)
Social network	−0.017 **	−0.017 **	0.019 **	0.014 **
	(0.007)	(0.007)	(0.008)	(0.006)
Social trust	−0.016 **	−0.016 **	0.018 **	0.013 **
	(0.007)	(0.007)	(0.008)	(0.006)
Social participation	−0.027 ***	−0.026 ***	0.031 ***	0.022 ***
	(0.007)	(0.006)	(0.007)	(0.006)
Social norms	−0.026 ***	−0.026 ***	0.030 ***	0.022 ***
	(0.006)	(0.006)	(0.007)	(0.005)
*Mianzi*	−0.015 ***	−0.015 ***	0.017 ***	0.012 ***
	(0.004)	(0.004)	(0.004)	(0.003)
Gender	0.005	0.005	−0.006	−0.004
	(0.013)	(0.013)	(0.015)	(0.011)
Age	0.001 *	0.001 *	−0.001 **	−0.001 *
	(0.001)	(0.001)	(0.001)	(0.001)
Education	0.011	0.011	−0.013	−0.010
	(0.008)	(0.008)	(0.009)	(0.007)
Political status	−0.030	−0.030	0.035	0.026
	(0.026)	(0.026)	(0.030)	(0.022)
Annual household income	0.003	0.003	−0.003	−0.003
	(0.006)	(0.006)	(0.007)	(0.005)
Total resident population	−0.004	−0.004	0.005	0.003
	(0.004)	(0.004)	(0.005)	(0.004)
Policy understanding	0.009	0.009	−0.010	−0.008
	(0.006)	(0.006)	(0.007)	(0.005)
Environmental awareness	−0.006	−0.006	0.007	0.005
	(0.007)	(0.007)	(0.008)	(0.006)
Hazard perception	−0.001	−0.001	0.001	0.001
	(0.006)	(0.006)	(0.007)	(0.005)
Waste-sorting facility	−0.082 ***	−0.081 ***	0.094 ***	0.069 ***
	(0.017)	(0.016)	(0.018)	(0.014)

Note: *, **, and *** donate a statistical significance at the 10%, 5%, 1%, respectively.

**Table 5:** Results of the moderating effect of *mianzi*.

Variables	Model (5) (All the Samples)	Model (6) (Low *mianzi*)	Model (7) (High *mianzi*)
Personal norms	0.110 ***	0.137 **	0.102 *
	(0.040)	(0.062)	(0.054)
Social network	0.099 **	0.070	0.120 *
	(0.039)	(0.051)	(0.061)
Social trust	0.092 **	0.081	0.123 **
	(0.042)	(0.067)	(0.055)
Social participation	0.157 ***	0.137 **	0.138 **
	(0.038)	(0.056)	(0.058)
Social norms	0.154 ***	0.143 ***	0.144 ***
	(0.035)	(0.049)	(0.053)
*Mianzi*	0.087 ***	—	—
	(0.021)		
Control variables	Controlled		
Log Likelihood	−1010.739	−444.596	−540.071
LR (P > chi^2^)	214.350 ***	49.220 ***	118.010 ***
Pseudo R^2^	0.096	0.053	0.099
Observations	943	441	502

Note: *, **, and *** donate a statistical significance at the 10%, 5%, 1%, respectively.

## Data Availability

The datasets used and/or analyzed during the current study are available from the corresponding author on reasonable request.
